# Behavioural and Noise-Level Differences Associated with Response-Independent Treat Delivery in Shelter Dogs

**DOI:** 10.3390/ani16193016

**Published:** 2026-09-25

**Authors:** Phil Vokins, Jacklyn Jaye Ellis

**Affiliations:** 1 Etobicoke Humane Society, 67 Six Point Road, Toronto, ON M8Z 2X3, Canada; 2 Faculty of Animal Science and Behavior, Unity Environmental University, 70 Farm View Drive, New Gloucester, ME 04260, USA; 3 Toronto Humane Society, 11 River Street, Toronto, ON M5A 4C2, Canada

**Keywords:** stress, kennels, barking, anticipatory, non-contingent, adopter preference, animal welfare

## Abstract

Animal shelters can be stressful environments for dogs. Undesirable in-kennel behaviours may reflect poor welfare and negatively affect adoption outcomes. Response-independent treat delivery has shown promise for improving in-kennel behaviour, but its effectiveness when delivered by multiple people is less understood. This study evaluated behavioural and noise-level differences associated with response-independent treat delivery in shelter dogs and responses to a subsequent Novel Person. Twenty-four dogs were observed during Baseline and Intervention phases. During Intervention, volunteers delivered treats when approaching kennels. Compared with Baseline, dogs in the Intervention phase were more likely to be at the front of the kennel, face forward, and stand, and were less likely to be at the back of the kennel, face backward, or exhibit prolonged barking. Behavioural changes across and within days occurred primarily during the Baseline. During the Novel Person comparison, several behaviours differed from Trial 4 during Baseline, while standing increased during Intervention. Because Baseline always preceded Intervention, phase was confounded with time, and the observed differences cannot be attributed unequivocally to treat delivery. The observed phase-associated differences in dogs’ in-kennel presentation appeared context-dependent and should not be interpreted as a generalized improvement in behaviour or welfare.

## Simple Summary

Animal shelters aim to provide good welfare for dogs while finding homes, but kennel environments can be stressful. This stress may affect how dogs behave when people walk past their kennel and may influence how adoptable they appear. One increasingly common practice is for staff, volunteers, or visitors to toss treats to dogs as they pass by, to create a positive association with people. This study examined behavioural and noise-level differences associated with treat delivery by a variety of passers-by in a shelter setting. Twenty-four dogs were observed during Baseline and Intervention phases. During Intervention, dogs were more often observed at the front of the kennel, facing forward, and standing, and were less often observed at the back of the kennel, facing backward, or barking for a prolonged period. No overall phase-associated difference was detected in noise levels, although maximum dB change differed between sets. Responses to a completely unfamiliar person also differed in some comparisons, but these findings were not unique to the Intervention phase. Overall, dogs behaved differently between the two phases, but these differences depended on the situation and did not necessarily mean that behaviour or welfare improved.

## 1. Introduction

Large numbers of dogs are surrendered or strayed each year, and are housed in animal shelters until adoption, or in some cases until their euthanasia [1].

The shelter is an inherently stressful environment for dogs. They may be separated from an attachment figure [2], have limited access to human interaction, experience a lack of control over their movement necessitated by kenneling, and have a limited ability to engage in species-specific behaviour such as exploring their environment [3]. This stress can negatively impact their welfare and contribute to the development or exacerbation of undesirable behaviours [4], often in the context of human presence [5]. These undesirable behaviours may cause the dogs to appear less adoptable to visitors [6], increasing their shelter residency time and intensifying the exhibition of these behaviours, creating a negative feedback loop.

Beyond the deleterious psychological stress of the shelter, the shelter can be physically stressful as well, particularly to dogs’ auditory senses. Noise levels in shelters can reach more than 125 decibels (dB) [7]. Scheifele et al. [8] found that, after six months in the shelter, all dogs tested showed measurable changes in hearing thresholds, suggesting hearing loss from chronic noise exposure.

Response-dependent treat delivery (giving a dog a treat when they perform a desired behaviour) has been shown to be an effective way to reduce undesirable in-kennel behaviours [9,10] and to reduce duration and continuous sound levels associated with barking [11] in the presence of humans. However, this protocol requires training and patience from the people performing it and it may therefore be difficult to achieve compliance from shelter staff and visitors. In-kennel response-independent treat delivery (giving a dog a treat irrespective of their behaviour) presents a promising strategy to help reduce behavioural indicators of stress and increase the presentation of adoptable behaviours in kenneled dogs in shelters because it is much more feasible, requiring little training or patience, and therefore requires fewer resources [10].

A number of studies have investigated the impact of response-independent treat delivery on in-kennel behaviour and noise volume. Protopopova and Wynne [10] found that response-independent treat delivery reduced the exhibition of undesirable behaviours (an aggregate measure comprising being located at the back of the kennel or out of sight, facing backwards, contact with kennel walls, and barking) as compared to a Baseline condition, and Gilchrist & Wynne [5] found a significant reduction in vocalizing in the tenth trial of response-independent treat delivery as compared to the first trial. Carrero & Bennett [12] found no significant difference in maximum dB readings between response-independent treat delivery and a Baseline condition, but they did report a reduction in dB over time in the response-independent treat delivery condition. Payne & Assemi [13] reported that response-independent treat delivery was associated with reduced mean dB as compared to a Baseline condition, and Zurlinden et al. [14] found a reduction in dB in the tenth trial of response-independent treat delivery as compared to the first trial—although neither Payne & Assemi [13] nor Zurlinden et al. [14] analyzed these differences statistically.

Although prior studies have reported behavioural and noise-level changes associated with response-independent treat-delivery protocols, several important gaps remain. First, while Intervention phases are typically compared to Baseline, no studies have statistically evaluated change over time. Carrero & Bennett [12] and Zurlinden et al. [14] reported changes in maximum dB across days but did not analyze these changes statistically. Gilchrist & Wynne [5] examined within-day behavioural changes, but their design limits interpretation. A 4-day Baseline phase with one trial per day was followed by a 10-day Intervention phase with 10 trials per day, and behaviour was compared between the first and last trial within sessions. Because the Baseline phase did not include multiple trials, within-session changes during Intervention may reflect repeated exposure (e.g., habituation, sensitization, or fatigue) rather than the effect of food delivery. Including comparable multi-trial Baseline sessions would have allowed these effects to be disentangled and enabled the evaluation of change both within and across days. Second, no studies have simultaneously assessed behaviours and noise level, limiting the understanding of whether these outcomes respond consistently. Finally, studies reporting behavioural effects [5,10] have implemented the protocol using a single researcher, leaving unclear how effective the Intervention would be when delivered by the range of staff, volunteers, and visitors—as it would realistically be implemented in a shelter setting.

Accordingly, the aim of the present study was to examine in-kennel behavioural and noise-level differences associated with response-independent treat delivery in shelter dogs. It was hypothesized that undesirable behaviours and noise level would decrease, and desirable behaviours would increase during the Intervention relative to Baseline phase; that these changes would be evident both across days and within days during the Intervention but not the Baseline phase; and that behavioural responses observed during Intervention would also be evident when dogs were approached by a Novel Person who had not previously participated in the protocol and was unfamiliar to the dog.

## 2. Materials and Methods

### 2.1. Study Site and Housing

This study was conducted at Toronto Humane Society (Toronto, ON, Canada) from August to September 2025. Dogs were housed in one of three kennel wards under standard shelter conditions. Each ward had access doors at both ends allowing for people to pass through and contained eight kennels, with a range of 2–4 dogs per ward across study days. Kennels ranged in total size from 4.9 m × 1.8 m × 2.8 m to 7.8 m × 2.4 m × 2.8 m (L × W × H) and consisted of two irregular shaped compartments connected with a guillotine door that facilitates separation when desirable (e.g., to facilitate the safe removal of food bowls). Each kennel had cement floors and cement walls and featured two doors. Each composed of a lower plexiglass panel and upper steel bars, which were staggered throughout wards to reduce direct visual dog–dog contact. The doors allow for human and dog entry and exit for operational purposes, and were not opened during any of the observations or in the same room while observations were being recorded.

Kennels consisted of two areas separated by a guillotine door, which remained open during data collection to allow full kennel access. Dogs were generally housed individually, except two co-housed dogs that entered the shelter together. Kennels contained a raised Kuranda bed, water and food bowls, toys, and soft bedding, unless contraindicated due to a history of ingestion or shredding of fabric items.

The shelter was closed to the public during data collection periods. Trials were scheduled such that dogs’ morning walks and meals were expected to be completed prior to the start of observations, thereby minimizing disruption to data collection; however, on some days, shelter operations ran behind schedule, and some dogs were removed from kennels for walks or other operational reasons such as medical checks and treatment during the trials.

### 2.2. Subjects

Twenty-four dogs participated overall. Not all dogs contributed complete datasets due to the shelter’s commitment to prioritizing adoption/foster matches, attending medical appointments, or shelter workflow priorities. In Set 1, 11 dogs contributed during Baseline and 6 during Intervention; 5 dogs contributed observations in both phases, while 7 contributed during only one phase. In Set 2, 11 dogs contributed during Baseline and 13 during Intervention; 10 dogs contributed in both phases and 4 during only one phase. Twelve unique dogs contributed to Set 1 and 14 to Set 2, with two dogs participating in both sets. Because dogs entered and left the study population during normal shelter operations, the number contributing observations varied by study day (Supplementary Table S1).

### 2.3. Experimental Design

The study comprised two 4-day phases (Baseline and Intervention) conducted in two sets. Because Baseline always preceded Intervention within each set, study phase was inherently confounded with time. Accordingly, phase-associated differences cannot be attributed uniquely to treat delivery, and the design should be interpreted as quasi-experimental rather than as a randomized or counterbalanced treatment comparison. In the first set, a 4-day Baseline phase was followed by a 3-day interval and then a 4-day Intervention phase. A 2-week period preceded the second set, which followed the same sequence. This period was included to increase the likelihood that different dogs would occupy the wards, thereby reducing the potential for carry-over learning; however, two dogs were present in both sets. The second set provided an additional observation period with a partly different shelter population, but was not treated as an independent replication because some dogs contributed to both sets and both sets followed the same fixed Baseline-to-Intervention sequence. Each test day, three volunteers—who were existing shelter volunteers with some but limited familiarity with the dogs and varied across study days—conducted four trials in each ward of kennels (approximately 10:00–11:00 AM). During both phases, volunteers mimicked potential adopters by visiting each dog for 20–30 s standing or crouching 1–3 feet from the kennel front, speaking softly, and refraining from touching the dogs. To promote consistency in behavioural recording, volunteers were emailed the protocols 1 week in advance for review, and, each day, a 30 min standardized training was provided, including purpose of experiment, expectations of the volunteers, and an opportunity for questions before data collection commenced. This period also included a demonstrated trial in a different room to those used for observations, supported by documented instructions for each volunteer to refer to if needed. A total of 26 volunteers participated, with some participating more than once to ensure adequate coverage on three occasions. Two volunteers participated on days in both Baseline and Intervention phases because of resourcing needs and their availability. Observer identity was recorded for each behavioural observation. Observers were not blinded to study phase because the presence or absence of treat delivery made the phase apparent during data collection. During the Baseline phase, volunteers did not deliver any treats and recorded the presence or absence of each of the 11 predefined behaviours at the end of each trial. During the Intervention phase, volunteers delivered one to three treats at the beginning of each trial, independent of dog behaviour, and recorded the presence or absence of the behaviours in the same manner as during the Baseline phase. Volunteers used discretion within this range and delivered a subsequent treat only if the preceding treat had been consumed. The exact number of treats delivered or consumed during each trial was not recorded. Most dogs received Benny Bully’s Liver Treats (Mississauga, ON, Canada), whereas dogs with medically indicated food restrictions received kibble from their regular diet. Accordingly, the protocol represented a pragmatic, real-world intervention rather than a completely standardized treatment. Treats were provided from buckets hung on the front of each kennel. Volunteers were asked to discontinue data collection for any dog showing negative reactions they judged intense enough that a typical adopter would move away, or which in their opinion was causing stress to the dog. There were no instances where this occurred during the experiments. A Novel Person (i.e., a different individual each day who had no prior interaction with the dogs) performed one trial immediately following the volunteer trials on each day. The Novel Person followed the protocols corresponding to the active phase (Baseline or Intervention). The Novel Person trial was conducted in the second set of phases only.

### 2.4. Behavioural Measures

Observers recorded the presence or absence (yes/no) of 11 predefined behavioural indicators (Table 1) during the 20–30 s observation window. Selected behaviours had previously been suggested as indicators of welfare, reactivity towards humans, or related to adopter preference [5,6,9,10,15,16,17,18].

### 2.5. Noise Monitoring

Three wall-mounted decibel monitors (Danoplus DP 366 sound level meter (manufactured in China)) recorded noise levels once per second during each data collection period. They have a measurement range of 30–130 dB with a stated accuracy of ±1.5 dB and calibration using the manufacturer’s user-manual steps was completed at the start of the experiment. Calibration was a manual process and each of the three metres used in the experiment were calibrated concurrently in the same room with consistent sound (recorded music) to ensure reliability and consistency. Sound levels were recorded from the hour before data collection until completion and were uploaded to spreadsheets by the researcher to correlate noise levels with human entry times into the kennel. The monitors were located in mesh baskets fixed to the wall near the centre of each kennel ward, with a small cloth towel surrounding the monitor body (but not the microphone) to mitigate against any vibrations caused by people or animals (Figure 1).

### 2.6. Comparisons of Interest and Statistical Analysis

Four comparisons were conducted to evaluate behavioural and noise-level changes: (1) differences between Baseline and Intervention phases; (2) differences between Day 1 and Day 4 within Baseline and Intervention phases; (3) differences between Trial 1 and Trial 4 within Baseline and Intervention phases; and (4) differences between Trial 4 and the Novel Person trial within Baseline and Intervention phases. The fourth comparison included only the second set of Baseline and Intervention phases because Novel Person data were collected only during Set 2. Novel Person observations always followed Trial 4; consequently, person type was structurally associated with observation order within the session.

No dogs were present in one ward on Day 3 of the Intervention phase in Set 2; therefore, no behavioural or dB data were collected for that ward-day. In addition, a dB monitor malfunctioned in a separate ward on Day 2 of the Intervention phase in Set 2, and no dB data from that ward-day were available for analysis.

Behavioural outcomes were analyzed separately for each of the 11 recorded behaviours using binomial generalized linear mixed-effects models with a logit link. Individual behavioural observations were retained as separate observations. Random effects were used to account for repeated observations of the same dog and, where estimable, repeated observations made by the same observer and repeated observations of the same dog on the same date.

For the Baseline-versus-Intervention comparison, models included phase, set, day within phase, trial, and ward as fixed effects. For Day 1 versus Day 4 comparisons, models included phase, day, the phase × day interaction, set, trial, and ward. For Trial 1 versus Trial 4 comparisons, models included phase, trial, the phase × trial interaction, set, day within phase, and ward. For Trial 4 versus Novel Person comparisons, analyses were restricted to Set 2 and to dog-dates with both a Novel Person observation and at least one Trial 4 observation. This yielded 51 matched dog-dates from 13 dogs; 7 Novel Person dog-dates without a corresponding Trial 4 observation were excluded. Models included phase, person type (Trial 4 or Novel Person), the phase × person-type interaction, day within phase, and ward. Observer was not included in Novel Person models because observer group was structurally associated with person type.

Several behaviours could not be analyzed inferentially in some comparisons because sparse observations or separation prevented reliable model estimation. These behaviours were summarized descriptively. Holm corrections were applied separately within each predefined family of behavioural tests. For the overall Baseline-versus-Intervention comparison, adjustment was applied across the nine estimable behaviours. For the Day 1 versus Day 4 and Trial 1 versus Trial 4 analyses, adjustments were applied separately across estimable within-Baseline contrasts, within-Intervention contrasts, and phase interaction tests. The same structure was used for Trial 4 versus Novel Person comparisons, with adjustment across the five estimable behaviours. For noise-level analyses, Holm correction was applied across the two outcomes, median and maximum dB, within each corresponding contrast family. Odds ratios (ORs), 95% confidence intervals (CIs), adjusted predicted probabilities, and Holm-adjusted *p*-values were calculated from fitted models.

For noise-level analyses, ward-level median and maximum dB values during the data collection period were treated as the unit of analysis. For the Baseline-versus-Intervention comparison, models included phase, set, day within phase, and ward as fixed effects, with date included as a random effect where supported by the data. For Day 1 versus Day 4 comparisons, models included phase, day, the phase × day interaction, set, and ward, with date included as a random effect where supported. For Trial 1 versus Trial 4 comparisons, median and maximum dB values were paired within ward and date, and Trial 4 minus Trial 1 differences were modelled as outcomes with phase, set, day within phase, and ward as predictors and date as a random effect. For Trial 4 versus Novel Person comparisons, Novel Person minus Trial 4 differences were similarly modelled with phase, day within phase, and ward as predictors and date as a random effect; set was omitted because these analyses included Set 2 only. Estimated marginal means or adjusted paired differences and corresponding 95% CIs were calculated for dB analyses.

As a sensitivity analysis addressing the two-set design, the primary Baseline-versus-Intervention models were refitted with a phase × set interaction. Baseline-to-Intervention differences were estimated separately within Set 1 and Set 2, and the phase × set interaction was used to assess whether the magnitude of the phase-associated differences varied between sets. For behavioural outcomes, Holm correction was applied across the nine estimable phase × set interaction tests. The same approach was applied to the two dB outcomes, with Holm correction applied across the median and maximum dB interaction tests.

Model convergence and singularity were assessed for mixed-effects models, and model-specific residual diagnostics were examined. For linear models, residual normality and residual spread were additionally assessed. Random-effect structures were simplified only when variance estimates indicated that a random effect was unsupported by the data. Missing observations were not imputed, and analyses were based on available data meeting the criteria for each comparison. All statistical tests were two-tailed. All analyses were conducted in R (version 4.5.1; R Foundation for Statistical Computing, Vienna, Austria).

## 3. Results

### 3.1. Differences Between the Baseline and Intervention Phases

Behavioural analyses were based on repeated observations from 24 unique dogs. Although the mixed-effects models accounted for repeated observations, the number of observations should not be interpreted as the number of independent experimental subjects. Relative to Baseline, dogs in the Intervention phase were more likely to be observed at the front of the kennel (OR = 83.1, 95% CI [19.0, 363], Holm-adjusted *p* < 0.001) and facing forward (OR = 20.8, 95% CI [5.02, 86.0], Holm-adjusted *p* < 0.001). Given the large ORs and wide CIs, adjusted predicted probabilities may provide a more interpretable indication of the absolute phase-associated differences. The predicted probability of front-of-kennel position was 77.4% (95% CI [44.8, 93.5]) during Baseline and 99.6% (95% CI [98.0, 99.9]) during Intervention; for facing forward, the corresponding probabilities were 96.4% (95% CI [91.3, 98.6]) and 99.8% (95% CI [99.1, 100.0]), respectively. Conversely, dogs were less likely to be observed at the back of the kennel (OR = 0.015, 95% CI [0.004, 0.059], Holm-adjusted *p* < 0.001) or facing backward (OR = 0.070, 95% CI [0.017, 0.279], Holm-adjusted *p* < 0.001). Standing was also more common during the Intervention phase (OR = 4.81, 95% CI [2.13, 10.9], Holm-adjusted *p* < 0.001), whereas barking (long period) occurred less frequently (OR = 0.068, 95% CI [0.011, 0.425], Holm-adjusted *p* = 0.016). No significant differences were detected between phases for barking (short period), jumping, or tail wagging following Holm correction. Pacing and leaning/rubbing occurred too infrequently for inferential analysis and are therefore presented descriptively (Table 2; Figure 2). No significant difference was detected in median dB between the Baseline and Intervention phases (adjusted mean difference = 0.27 dB, 95% CI [−5.48, 6.03], Holm-adjusted *p* = 0.918). Maximum dB was lower during the Intervention phase, but this difference was not statistically significant (adjusted mean difference = −2.49 dB, 95% CI [−5.92, 0.94], Holm-adjusted *p* = 0.300) (Table 2; Figure 2).

Sensitivity analyses examining the Baseline-to-Intervention difference separately by set showed that the direction of behavioural differences was consistent between Set 1 and Set 2 for all nine behaviours analyzed inferentially. Although estimated differences varied between sets, no phase × set interactions were statistically significant after Holm correction (all adjusted *p* = 1.000). Median dB was also highly consistent between sets, with no significant phase × set interaction (Holm-adjusted *p* = 0.995). In contrast, the Baseline-to-Intervention change in maximum dB differed between sets: maximum dB decreased in Set 1 but not in Set 2, and the phase × set interaction was statistically significant after Holm correction (*p* = 0.022; Supplementary Table S2).

### 3.2. Differences Between the Day 1 and Day 4 Baseline and Intervention Phases Separately

#### 3.2.1. Baseline Phase

Compared with Day 1, barking (long period) was less likely to be observed on Day 4 during the Baseline phase (OR = 0.016, 95% CI [0.001, 0.282], Holm-adjusted *p* = 0.033). No significant differences were detected between Day 1 and Day 4 for barking (short period), front of kennel, back of kennel, jumping, standing, or tail wagging following Holm correction. Facing forward, facing backward, pacing, and leaning/rubbing could not be reliably analyzed inferentially because of sparse observations or separation and are therefore presented descriptively. There was no significant difference between Day 1 and Day 4 in either median dB (adjusted mean difference = 6.48 dB, 95% CI [−16.1, 29.1], Holm-adjusted *p* = 0.857) or maximum dB (adjusted mean difference = −3.15 dB, 95% CI [−16.3, 10.0], Holm-adjusted *p* = 0.857) (Table 3; Figure 3).

#### 3.2.2. Intervention Phase

No significant differences were detected between Day 1 and Day 4 for any of the seven behaviours analyzed inferentially: barking (short period), barking (long period), front of kennel, back of kennel, jumping, standing, or tail wagging. Facing forward, facing backward, pacing, and leaning/rubbing are presented descriptively because of sparse observations or separation. Similarly, there was no significant difference between Day 1 and Day 4 in either median dB (adjusted mean difference = 0.10 dB, 95% CI [−22.5, 22.7], Holm-adjusted *p* = 1.000) or maximum dB (adjusted mean difference = 2.85 dB, 95% CI [−10.3, 16.0], Holm-adjusted *p* = 1.000) (Table 3; Figure 3).

No significant phase × day interactions were detected for any of the seven behaviours analyzed inferentially. Similarly, the phase × day interaction was not significant for either median dB (adjusted difference in change = −6.38 dB, 95% CI −38.3 to 25.6, Holm-adjusted *p* = 0.760) or maximum dB (adjusted difference in change = 6.00 dB, 95% CI −12.6 to 24.6, Holm-adjusted *p* = 0.760).

### 3.3. Differences Between Trial 1 and Trial 4 for Baseline and Intervention Phases Separately

#### 3.3.1. Baseline Phase

Compared with Trial 1, observations from Trial 4 were less likely to include barking (long period) (OR = 0.071, 95% CI [0.025, 0.202], Holm-adjusted *p* < 0.001), front of kennel (OR = 0.082, 95% CI [0.031, 0.214], Holm-adjusted *p* < 0.001), jumping (OR = 0.116, 95% CI [0.032, 0.420], Holm-adjusted *p* = 0.004), facing forward (OR = 0.271, 95% CI [0.118, 0.622], Holm-adjusted *p* = 0.006), standing (OR = 0.110, 95% CI [0.054, 0.224], Holm-adjusted *p* < 0.001), and tail wagging (OR = 0.115, 95% CI [0.054, 0.243], Holm-adjusted *p* < 0.001). Conversely, dogs were more likely to be observed at the back of the kennel in Trial 4 than Trial 1 (OR = 10.9, 95% CI [4.36, 27.3], Holm-adjusted *p* < 0.001). No significant differences were detected between trials for barking (short period) or facing backward following Holm correction. Pacing and leaning/rubbing were too sparse for reliable inferential modelling and are therefore presented descriptively. Median dB was significantly lower in Trial 4 than Trial 1 (adjusted paired difference = −4.55 dB, 95% CI [−8.02, −1.08], Holm-adjusted *p* = 0.031). Maximum dB was also significantly lower in Trial 4 (adjusted paired difference = −11.62 dB, 95% CI [−20.5, −2.69], Holm-adjusted *p* = 0.031) (Table 4; Figure 4).

#### 3.3.2. Intervention Phase

No significant differences were detected between Trial 1 and Trial 4 for any of the nine behaviours analyzed inferentially. Pacing and leaning/rubbing are therefore presented descriptively. Similarly, there was no significant difference between Trial 1 and Trial 4 in either median dB (adjusted paired difference = −1.47 dB, 95% CI [−4.96, 2.01], Holm-adjusted *p* = 0.550) or maximum dB (adjusted paired difference = −4.65 dB, 95% CI [−13.6, 4.31], Holm-adjusted *p* = 0.550) (Table 4; Figure 4).

Significant phase × trial interactions were detected for front of kennel, back of kennel, and standing, indicating that Trial 1-to-Trial 4 changes in these behaviours differed between phases. No significant phase × trial interactions were detected for the remaining inferentially analyzed behaviours. Despite the significant within-Baseline reductions in median and maximum dB, Trial 1-to-Trial 4 change did not differ significantly between phases for either median dB (difference in change = 3.08 dB, 95% CI [−1.84, 8.00], Holm-adjusted *p* = 0.387) or maximum dB (difference in change = 6.97 dB, 95% CI [−5.68, 19.6], Holm-adjusted *p* = 0.387).

### 3.4. Differences Between Trial 4 and Novel Person Trial for Baseline and Intervention Phases Separately (Second Set of Baseline and Intervention Phases Only)

#### 3.4.1. Baseline Phase

Compared with Trial 4, dogs were more likely to be observed at the front of the kennel during the Novel Person trial (OR = 40.2, 95% CI [2.13, 761], Holm-adjusted *p* = 0.041) and to exhibit tail wagging (OR = 8.19, 95% CI [1.73, 38.7], Holm-adjusted *p* = 0.040). Conversely, dogs were less likely to be observed at the back of the kennel during the Novel Person trial (OR = 0.057, 95% CI [0.006, 0.501], Holm-adjusted *p* = 0.040). No significant differences were detected between Trial 4 and the Novel Person trial for barking (short period) or standing following Holm correction. Barking (long period), facing forward, facing backward, pacing, leaning/rubbing, and jumping could not be reliably analyzed inferentially because of sparse observations or separation and are therefore presented descriptively. There was no significant difference between Trial 4 and the Novel Person period in either median dB (adjusted paired difference = 3.78 dB, 95% CI [−9.17, 16.7], Holm-adjusted *p* = 0.789) or maximum dB (adjusted paired difference = −2.42 dB, 95% CI [−33.4, 28.6], Holm-adjusted *p* = 0.815) (Table 5; Figure 5).

#### 3.4.2. Intervention Phase

Compared with Trial 4, standing was more likely to be observed during the Novel Person trial (OR = 6.49, 95% CI [1.93, 21.8], Holm-adjusted *p* = 0.013). No significant differences were detected between Trial 4 and the Novel Person trial for barking (short period), front of kennel, back of kennel, or tail wagging following Holm correction. Barking (long period), facing forward, facing backward, pacing, leaning/rubbing, and jumping are therefore presented descriptively. There was no significant difference between Trial 4 and the Novel Person period in either median dB (adjusted paired difference = 0.84 dB, 95% CI [−11.25, 12.9], Holm-adjusted *p* = 1.000) or maximum dB (adjusted paired difference = −1.84 dB, 95% CI [−32.0, 28.3], Holm-adjusted *p* = 1.000) (Table 5; Figure 5).

No significant phase × person-type interactions were detected for any of the five inferentially analyzed behaviours. Similarly, Trial 4-to-Novel Person change did not differ significantly between phases for either median dB (difference in change = −2.94 dB, 95% CI [−20.5, 14.6], Holm-adjusted *p* = 1.000) or maximum dB (difference in change = 0.57 dB, 95% CI [−42.6, 43.7], Holm-adjusted *p* = 1.000).

## 4. Discussion

This study examined behavioural and noise-level differences associated with response-independent treat delivery in shelter dogs across phases, across days, within days, and in response to a Novel Person. In the overall Baseline-versus-Intervention comparison, dogs were more likely, during Intervention, to be observed at the front of the kennel, facing forward, and standing, and were less likely to be observed at the back of the kennel, facing backward, or barking for a prolonged period. However, temporal analyses provided a more complex picture. Several behavioural and noise-level changes occurred during the Baseline phase, whereas fewer within-phase changes were detected during Intervention. Novel Person responses also differed from Trial 4 for several behaviours, but the pattern varied by phase. Taken together, the findings indicate substantial phase-associated behavioural differences, but do not provide unequivocal evidence that response-independent treat delivery produced generalized improvements across behaviours, time, or contexts. The behavioural changes observed during Baseline, but not Intervention, may reflect reduced responding to repeated human approaches over time; possible explanations include habituation, learned irrelevance, or other temporal processes.

In the overall comparison between Baseline and Intervention phases, several behavioural outcomes differed in the hypothesized direction: kennel position (front or back half) and kennel orientation (facing forwards or backwards). Facing forward was more likely during the Intervention phase than during Baseline. These changes may reflect dogs being more attentive in response to anticipated food delivery or another outcome of interest. It is also possible that the treat placement at the front of the kennel required the dog to come to the front of the kennel and face forwards in order to consume it. In both cases, these outcomes of attentiveness and body position are not necessarily indicative of positive affective state or improved welfare but have been shown to be linked to adopter selection [6]. Standing also increased, a behaviour Protopopova et al. [15] found to be exhibited more frequently in dogs with a longer length of stay and therefore considered it an undesirable behaviour from the perspective of an adopter. However, Wells [19] found adopters more likely to select a dog who is standing, so the relationship between this behaviour and adoption is not certain, and its interpretation may not be straightforward.

Comparison of behaviours from Day 1 to Day 4 and Trial 1 to Trial 4 in each phase produced initially surprising results. It was hypothesized that desirable behaviours would increase and undesirable behaviours would decrease, both across and within days in the Intervention phase. However, no significant within-phase behavioural changes were detected during Intervention in either temporal comparison. During Baseline, long-period barking decreased significantly from Day 1 to Day 4. Within days, Trial 4 was associated with decreases in long-period barking, front-of-kennel location, jumping, facing forward, standing, and tail wagging, and an increase in back-of-kennel location relative to Trial 1. Importantly, however, phase × trial interactions were significant only for front-of-kennel location, back-of-kennel location, and standing, and no significant phase × day interactions were detected. Thus, evidence that temporal patterns differed between Baseline and Intervention was limited to these three within-day behaviours. One possible explanation for some of the behavioural changes observed during Baseline is reduced responding to repeated human approaches when these approaches were not followed by an outcome of interest to the dog. This could be consistent with processes such as learned irrelevance, habituation, or extinction, which cannot be distinguished by the present design. Dogs may initially have responded to approaching people because human presence could predict social interaction, a walk, food, or another valued outcome. Repeated approaches without such an outcome could subsequently reduce some forms of attention or anticipatory responding. Experimental evidence in dogs indicates that delaying or withholding an expected reward leads to frustration-related behavioural and physiological responses [20,21], while learning theory more generally predicts that conditioned responding can decline when expected reinforcement is repeatedly absent [22]. Descriptively, long-period barking and pacing on Day 1 of the Intervention phase appeared more similar to Day 4 than Day 1 of Baseline. Although this pattern could be consistent with carry-over of prior experience, it was not formally tested and should be interpreted cautiously. Likewise, the absence of detectable Trial 1-to-Trial 4 behavioural changes during Intervention could reflect the early delivery of treats reducing opportunities for continued anticipation or frustration, but the present data do not establish this mechanism.

Comparison of the behaviours between Trial 4 and the Novel Person in the Baseline and Intervention phases separately also produced surprising results. During Baseline, dogs were more likely to be observed at the front of the kennel and tail wagging, and less likely to be observed at the back of the kennel, during the Novel Person observation than during Trial 4. During Intervention, standing was more likely during the Novel Person observation, while no significant differences were detected for the other behaviours analyzed inferentially. Importantly, no phase × person-type interactions were significant, indicating that the Trial 4-to-Novel Person changes did not differ significantly between Baseline and Intervention for the behaviours that could be analyzed inferentially. The Novel Person observations also always occurred after Trial 4, so these differences cannot be attributed uniquely to the novelty of the person. Several mechanisms could plausibly contribute to these findings. Repeated treat-associated human approaches during Intervention may have altered dogs’ expectations of approaching people, and novelty can interact with reward-related and attentional processes [23,24,25,26,27,28]. However, the present behavioural data do not establish that the increased standing observed during Intervention reflected a positive affective state or positive reward anticipation. It could also reflect heightened arousal, vigilance, frustration, increased food motivation, or another response to the unfamiliar person or to their later position within the session. Likewise, the behavioural differences observed during Baseline demonstrate that responding to the Novel Person was not unique to the Intervention phase. In addition, the Novel Person analyses were based on a substantially smaller matched sample than the primary phase comparison (51 matched dog-dates from 13 dogs). The resulting wide confidence intervals indicate limited precision and suggest caution when interpreting both statistically significant findings and the absence of detectable differences. Independent affective or physiological measures, together with counterbalancing of observation order, would be required to determine the mechanisms underlying these responses with greater confidence.

Noise-level findings were more limited. Neither median nor maximum dB differed significantly between the Baseline and Intervention phases overall, and neither changed significantly from Day 1 to Day 4 within either phase. Within days, however, both median and maximum dB were significantly lower in Trial 4 than Trial 1 during Baseline, whereas no significant Trial 1-to-Trial 4 differences were detected during Intervention. Importantly, the corresponding phase × trial interactions were not significant, so the magnitude of within-day change did not differ statistically between phases. Similarly, neither median nor maximum dB differed significantly between Trial 4 and the Novel Person observation in either phase, and no phase × person-type interactions were detected. Sensitivity analyzes comparing the Baseline-to-Intervention difference between sets showed that median dB was highly consistent across Set 1 and Set 2, whereas maximum dB was less stable: maximum dB decreased during Intervention in Set 1 but not Set 2, resulting in a significant phase × set interaction. The inconsistency between sets suggests that any association between the protocol and maximum noise levels may be context-dependent and was not robust across the two study periods. The findings in this paper mirror those of Carrero & Bennett [12], who also found no significant difference in maximum dB between response-independent treat delivery and a Baseline condition. Both Carrero & Bennett [12] and Zurlinden et al. [14] reported reductions in dB across days—although they did not analyse these changes statistically. One potential explanation for the relative lack of significant differences in dB measurements from all comparisons in the current study is that noise levels were already somewhat lower than in other studies: Payne & Assemi [13] reported a mean dB of 85.1 in their Baseline phase and 68.1 in their Intervention period, while the current study reports a median dB of 56.71 (mean = 61.14) for the Baseline phase and a median dB of 56.29 (mean = 60.12) for the Intervention phase. The relatively low noise levels may have limited the magnitude of additional reductions that could be observed. These reduced noise levels could be explained by the glazing on the lower half of THS kennel doors—which Beesley and Mills [29] found to result in fewer barking bouts—or the small number of dogs per ward—although Zurlinden et al. [14] found the number of dogs barking did not necessarily increase the overall dB. Finally, as Zurlinden et al. [14] also noted, dB are measured on a logarithmic scale, so an increase of 10 dB on a linear scale actually represents a multiplication of ten times the intensity of actual sound.

This study has a number of limitations. Firstly, Baseline always preceded Intervention within each set, so phase was confounded with time. Although the statistical models adjusted for measured temporal factors such as set, day within phase, trial, and ward, they cannot distinguish phase-associated differences potentially related to treat delivery from those arising from other changes occurring over time, including habituation to the shelter environment, repeated exposure to people, changes in shelter operations, or changes in the dogs contributing data. Relatedly, the shelter population changed throughout the study as dogs were adopted, fostered, attended medical appointments, or were otherwise unavailable. Within-day changes, such as the removal of a dog for operational reasons, were not recorded because of their unpredictable timing. Twenty-four unique dogs participated overall, with two dogs participating in both sets. This turnover may have influenced phase comparisons if dogs entering or leaving the study differed systematically in behaviour or noise contribution. Although the mixed-effects models accounted for repeated observations, the independent sample remained limited to 24 dogs. The large number of behavioural observations therefore should not be interpreted as a correspondingly large number of independent experimental subjects. The wide confidence intervals around several estimates, including the large odds ratios for front-of-kennel position and forward orientation, indicate limited precision. The Set 1 versus Set 2 sensitivity analysis provided some reassurance that behavioural differences and median dB were broadly consistent across sets, although the maximum dB difference varied significantly between sets, indicating that this outcome was less stable. Second, the treat delivered was not fully standardized. Most dogs received liver treats, whereas dogs with dietary restrictions received their regular kibble or other permitted food, which necessarily introduced heterogeneity of treat reward and may have made treat-delivery less valuable and/or novel. The number of dogs receiving each food type was not recorded, so possible differences in food value or responsiveness could not be examined. Likewise, volunteers were instructed to deliver one to three treats at the beginning of each Intervention trial, but the exact number delivered and whether all treats were consumed were not systematically recorded. This pragmatic flexibility increased the protocol’s relevance to routine shelter operations but may also have contributed to heterogeneity in behavioural responses by varying the strength of the food-associated experience across dogs and observations. Third, there was a two-week gap between running the initial set of Baseline and Intervention phases and the second set in hopes of animal flow resulting in a different population of dogs, but two dogs were present in the same wards in both sets, and their experience during the first set may have influenced their behaviour during the second. Fourth, behavioural outcomes were recorded as binary presence/absence measures within each trial. This approach may have limited sensitivity to detect more subtle changes in behavioural expression. Measures of behavioural frequency, duration, or intensity may have provided a more detailed understanding of changes across conditions. Finally, the presence or absence of behaviours was coded live instead of via video recording. This decision was made due to practical resource limitations. Although observers received standardized training intended to promote consistency, inter-observer reliability was not formally assessed. Standardized training may have promoted consistency among observers, but it does not substitute for formal assessment of inter-observer agreement. The lack of video also prevented the retrospective assessment of measurement precision and may have limited the ability to record multiple behaviours occurring simultaneously.

There may be practical limitations with implementing the procedure on an ongoing basis. There will be a financial cost of buckets and treats and the time required to fill them. An increase in the number of dogs with dietary restrictions may also increase the daily time required to fill those buckets, which can be the largest hurdle in implementing any new programme in shelter environments. Encouraging compliance from passers-by—whether they are staff, volunteers, or members of the public—may also be an obstacle.

The findings of the current study reveal several interesting ideas for future research. Previous work by Carrero and Bennett [12] demonstrated that compliance with participation protocols among kennel passers-by was significantly associated with noise levels, with each one-percentage-point increase in compliance corresponding to an average decrease of 17.3 dB in maximum volume. Future research could extend this work by examining how variation in protocol compliance relates not only to noise levels but also to behavioural expression, or compare response-independent treat delivery with alternative practical shelter-based interventions. Further work should also consider more detailed behavioural measurement. For example, differentiating bark types or other vocalizations may help clarify both the motivation underlying vocal behaviour and its potential influence on adopter perceptions. Similarly, coding features of tail wagging, rather than its presence alone, may provide more informative behavioural data. Future studies could also compare response-independent treat delivery with other practical shelter protocols. For example, automated treat delivery could separate food presentation from human approach, while varying the location of treat delivery could help determine whether front-of-kennel behaviour reflects food location rather than a broader change in human-directed behaviour. Response-dependent reinforcement of specific behaviours could also be compared with response-independent delivery. More controlled designs could further examine the potential roles of social facilitation, novelty, and goal frustration in shaping dogs’ responses across repeated human approaches. Finally, video analyses of dog behaviour during real adopter interactions could be undertaken to compare the behaviours of dogs that are selected for adoption with those that are not, providing valuable information on the traits that may influence adopter decision-making.

## 5. Conclusions

This study identified several behavioural differences between the Baseline and Intervention phases of response-independent treat delivery in shelter dogs. During Intervention, dogs were more likely to be observed at the front of the kennel, facing forward, and standing, and were less likely to be observed at the back of the kennel, facing backward, or barking for a prolonged period. However, behavioural changes across and within days were limited and occurred primarily during Baseline, and the Novel Person findings did not provide evidence that behavioural responses generalized uniquely during Intervention. Noise levels also showed little overall phase-associated change. Median dB was consistent across study sets, whereas maximum dB showed a set-specific difference, indicating less stable results for that outcome. Because Baseline always preceded Intervention, phase was confounded with time, and these differences cannot be attributed unequivocally to treat delivery. In addition, several behaviours may reflect attention, food anticipation, arousal, or kennel position rather than improved welfare or adopter appeal specifically. The observed phase-associated differences in dogs’ in-kennel presentation therefore appear context-dependent and should not be interpreted as a generalized improvement in behaviour or welfare. Future studies using counterbalanced designs, more detailed behavioural and affective measures, and controlled Novel Person comparisons would help clarify the mechanisms and practical value of this approach.

## Figures and Tables

**Figure 1:**
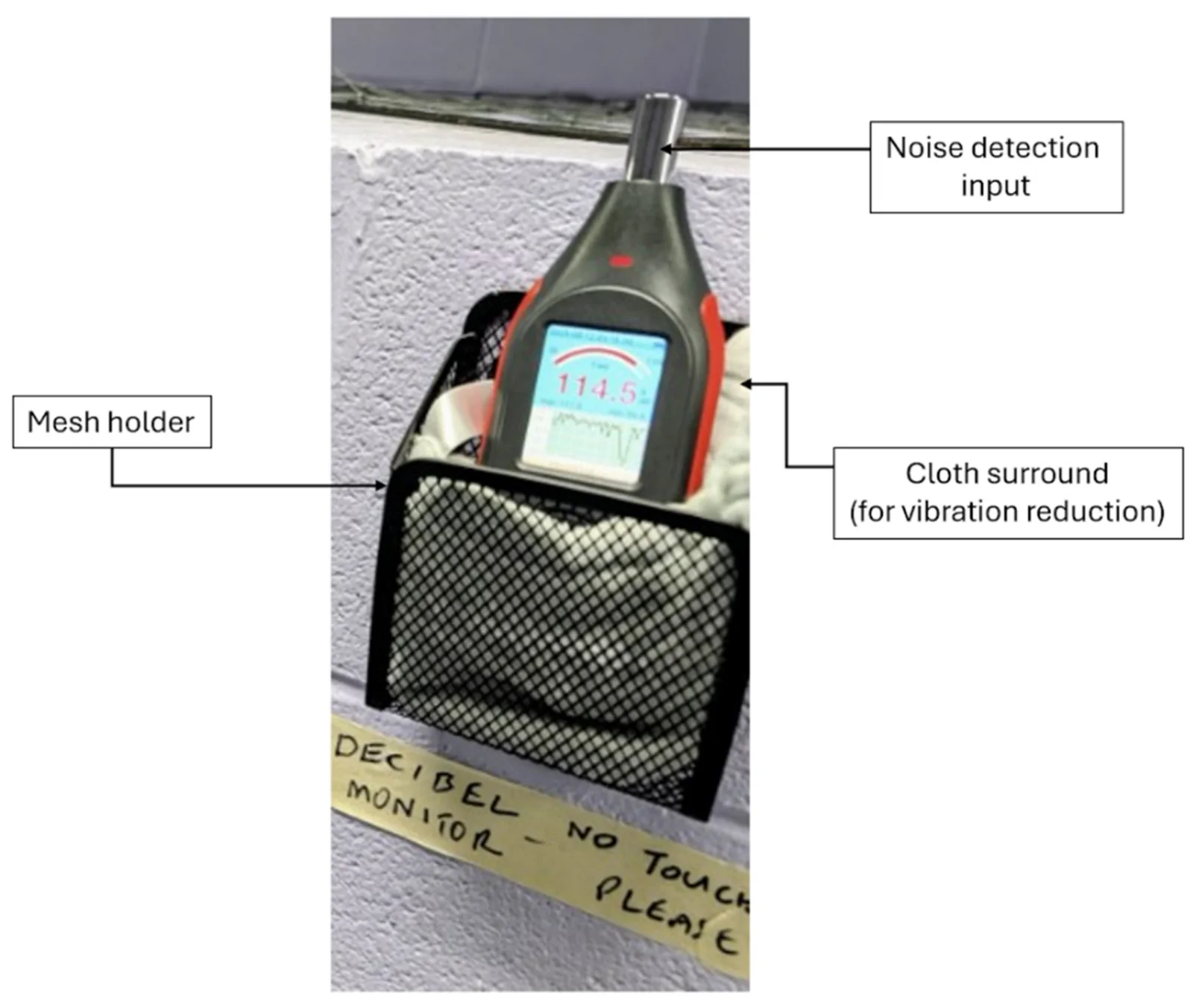
Decibel monitor installation.

**Figure 2:**
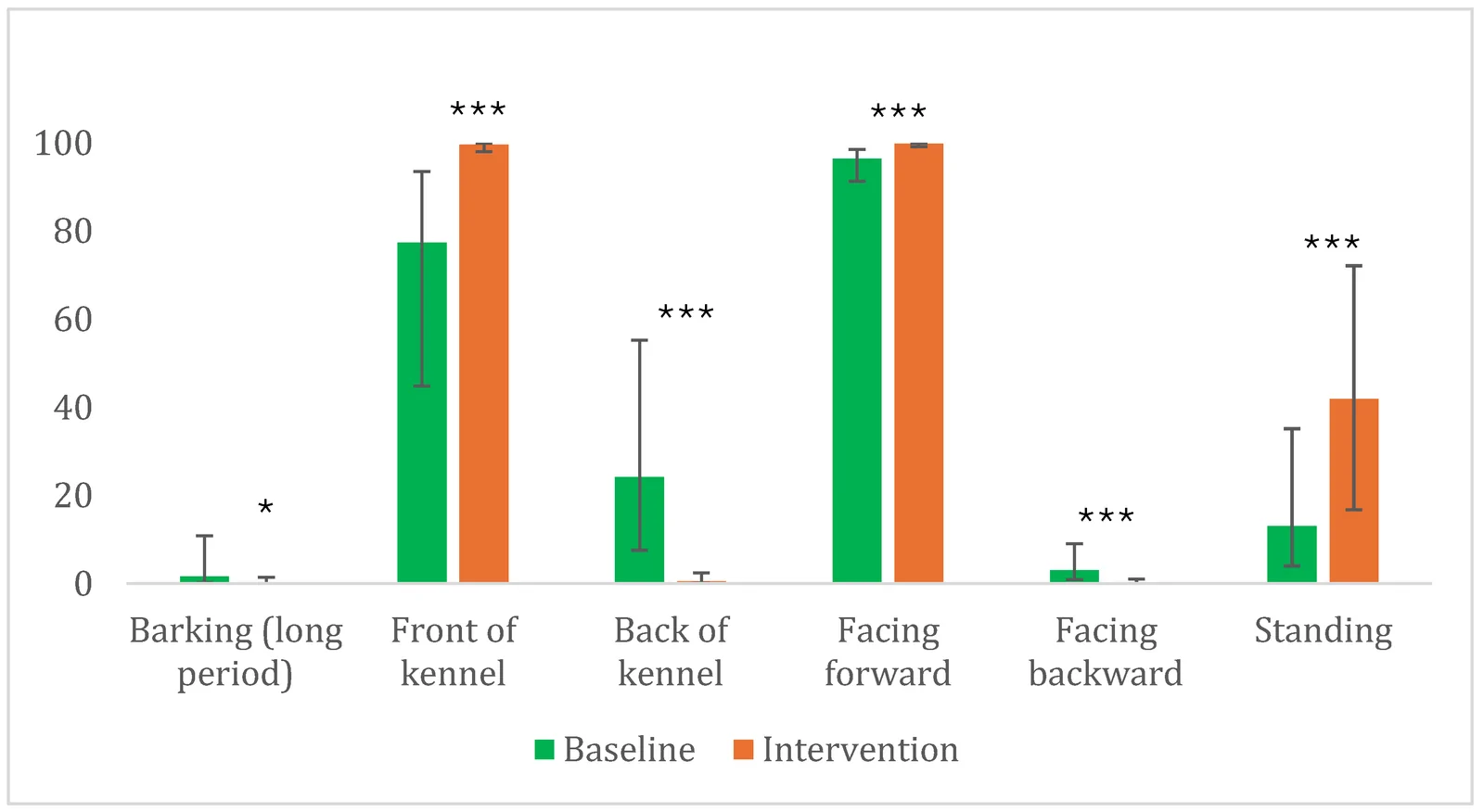
Model-adjusted predicted probabilities (%) and 95% CIs for behaviours during the Baseline and Intervention phases. Only behaviours with a statistically significant Baseline–Intervention difference are depicted. Statistical significance is based on Holm-adjusted *p*-values and is indicated as follows: * *p* < 0.05, *** *p* < 0.001.

**Figure 3:**
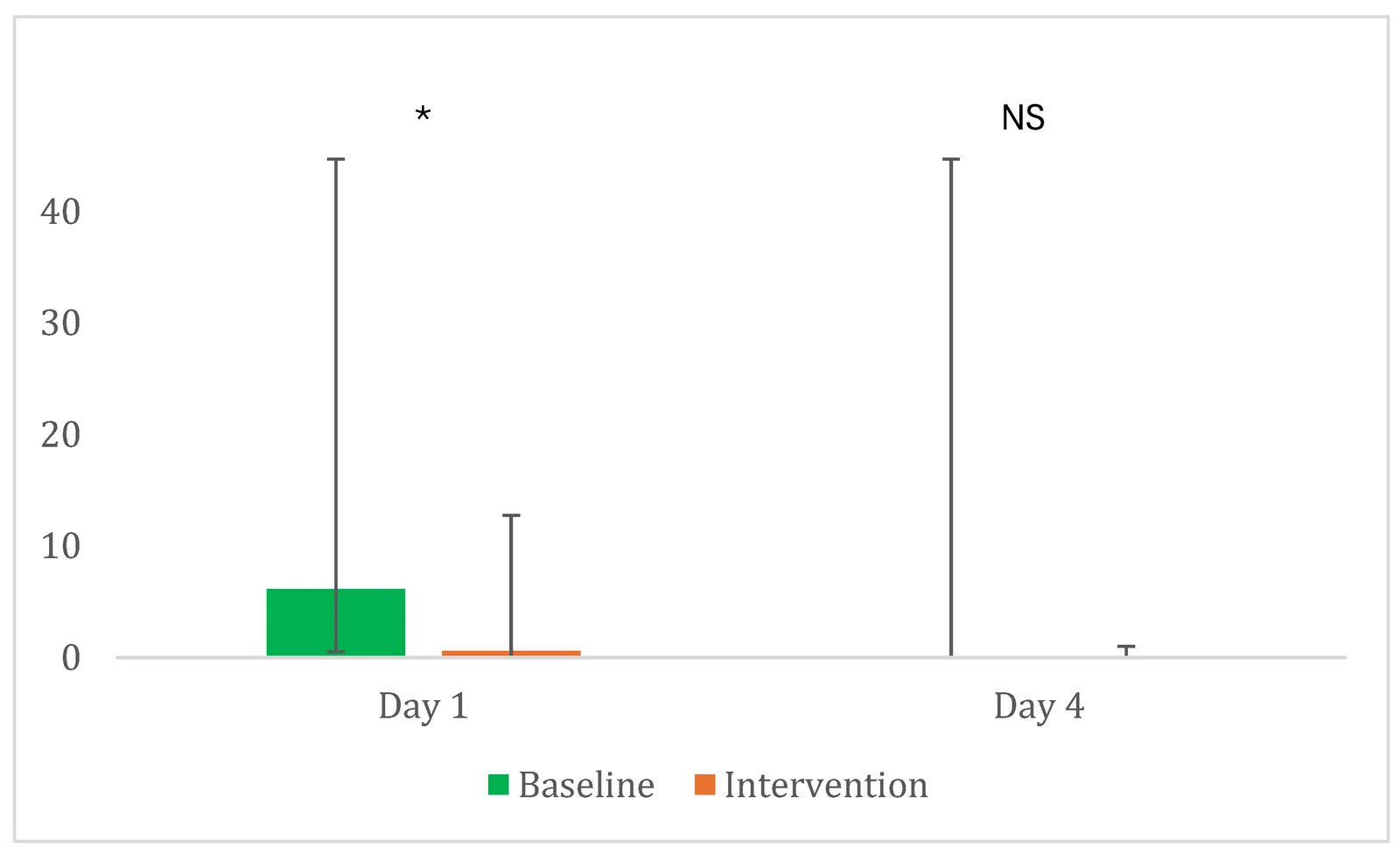
Model-adjusted predicted probability (%) and 95% CI of observations containing behaviours in Day 1 and Day 4, presented separately for Baseline and Intervention phases. Only behaviours with a statistically significant Day 1-to-Day 4 difference in at least one phase are depicted. Statistical significance is based on Holm-adjusted *p*-values and is indicated as follows: NS = not significant, * *p* < 0.05.

**Figure 4:**
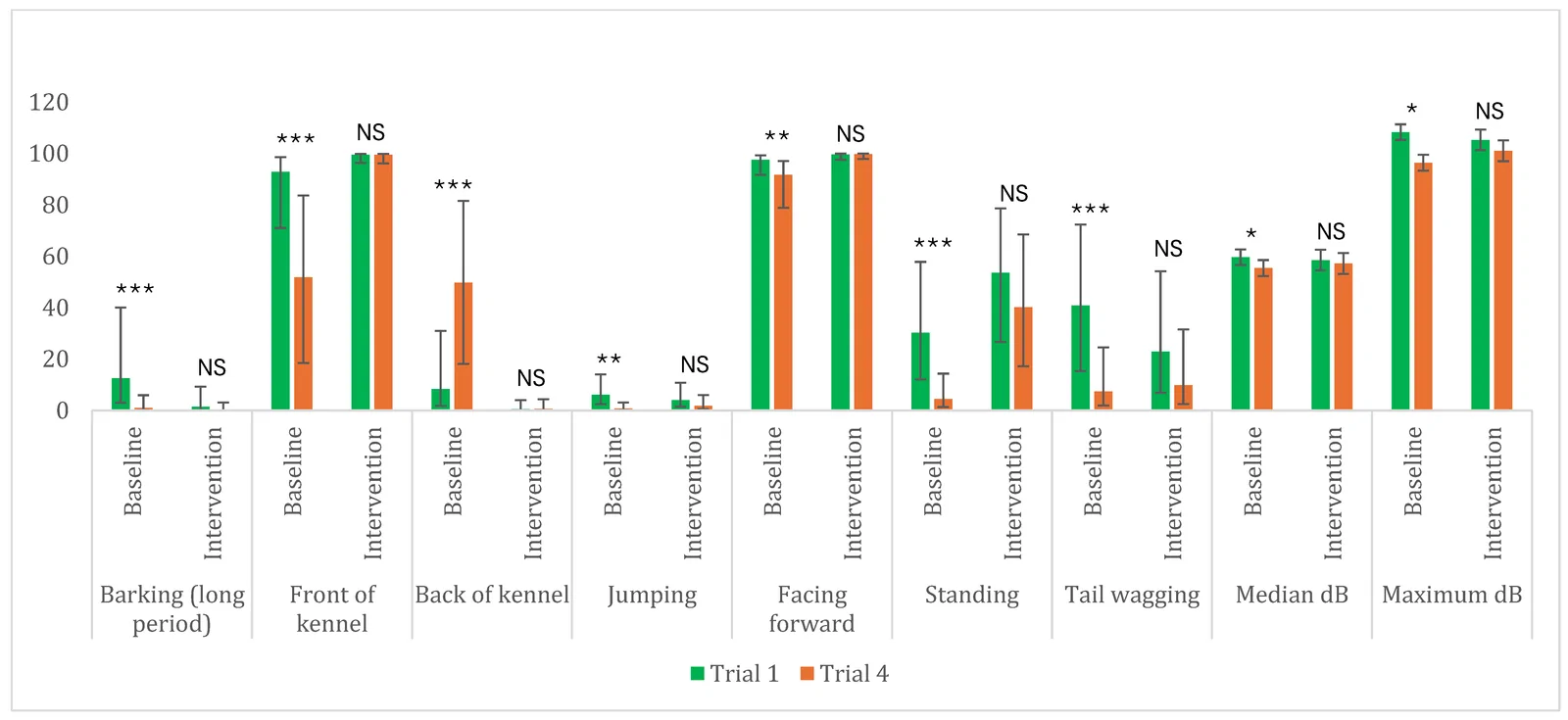
Comparison of Trial 1 and Trial 4 outcomes, presented separately for the Baseline and Intervention phases. Behaviour outcomes are shown as model-adjusted predicted probabilities (%) with 95% CIs, and ward-level noise outcomes are shown as model-adjusted mean median and maximum dB levels with 95% CIs. Only outcomes with a statistically significant within-phase Trial 1-to-Trial 4 difference in at least one phase are depicted. Statistical significance is based on Holm-adjusted *p*-values and is indicated as follows: NS = not significant, * *p* < 0.05, ** *p* < 0.01, *** *p* < 0.001.

**Figure 5:**
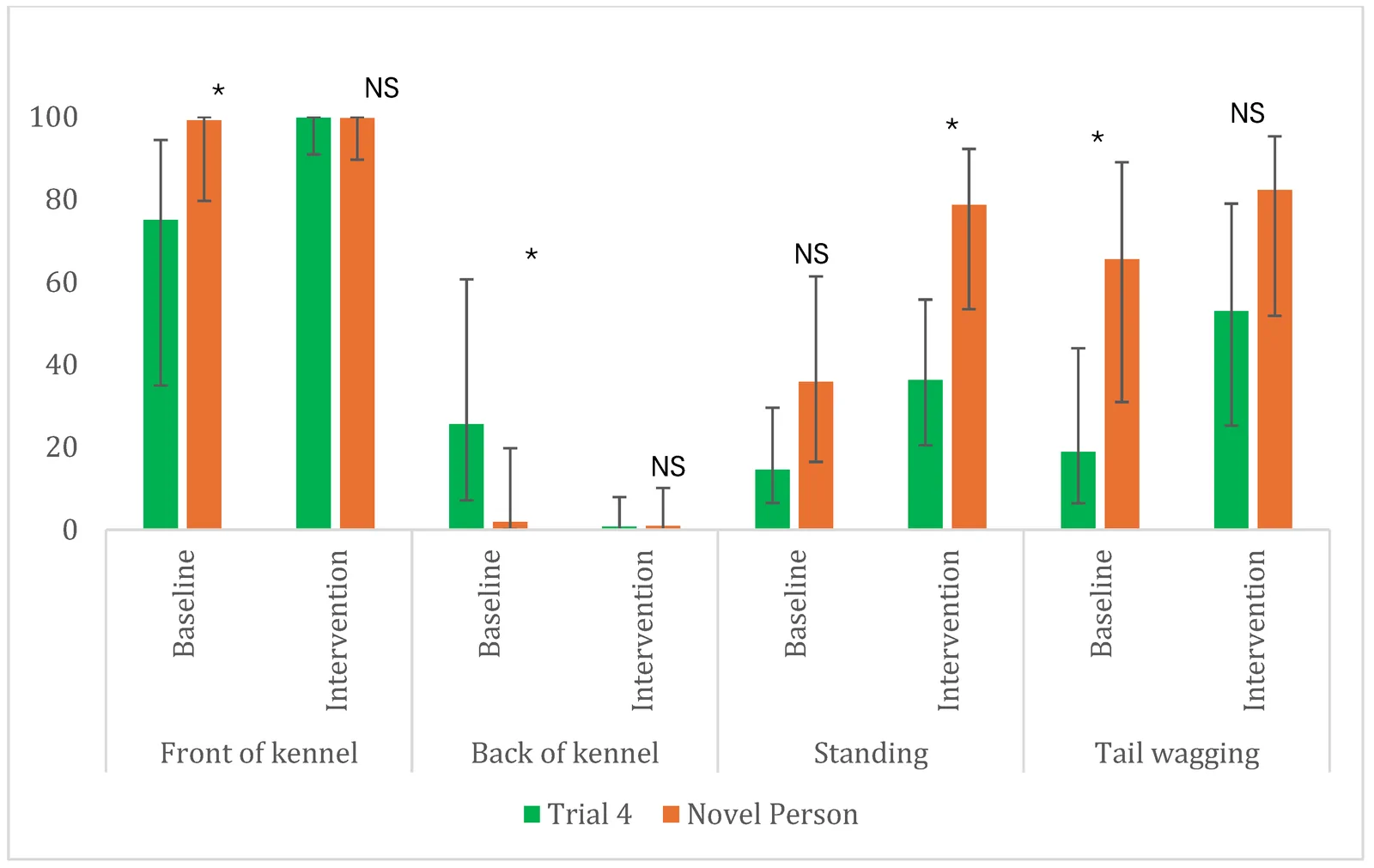
Model-adjusted predicted probability (%) and 95% CI of observed behaviours during Trial 4 and the Novel Person observation, presented separately for the Baseline and Intervention phases in Set 2. Only behaviours with a statistically significant within-phase Trial 4-to-Novel Person difference in at least one phase are depicted. Statistical significance is based on Holm-adjusted *p*-values and is indicated as follows: NS = not significant, * *p* < 0.05.

**Table 1:** Behaviours and operational definitions.

Behaviour	Operational Definition
Barking (short period)	Dog emits a bark, howl, or yelp (less than 3 s)
Barking (long period)	Dog emits bark, howl, or yelp (more than 3 s with <3 s between barks)
Front of kennel	Located at front of kennel, up to and including midpoint (in last 15 s of interaction)
Back of kennel	Dog is located behind the midpoint of the kennel or out of sight (in last 15 s of interaction)
Pacing	Repeatedly (>3) walk back and forth in fixed route
Leaning/rubbing on enclosure (excludes door)	Prolonged (>1 s) contact with the cage wall by pushing body side against kennel wall
Jumping	Both front paws make contact with the kennel door
Facing forward	Head is oriented such that you observer can see more than the side profile of face (at any point > 3 s in last 15 s of interaction)
Facing backward	No eyes are visible (>3 s in last 15 s of interaction)
Standing	Standing upright on all four legs > 3 s in last 15 s of interaction
Tail wagging	Continuous (>10 s) side to side tail movement

**Table 2:** Differences between Baseline and Intervention phases.

**Behaviour**	**Baseline** **Adjusted Probability (%)**	**Intervention** **Adjusted Probability (%)**	**OR (Intervention vs. Baseline)**	**95% CI**	**Holm-Adjusted *p*-Value**
Barking (short period)	5.7	3.2	0.55	0.26–1.14	0.219
Barking (long period)	1.6	0.1	0.068	0.011–0.425	0.016
Front of kennel	77.4	99.6	83.1	19.0–363	<0.001
Back of kennel	24.2	0.5	0.015	0.004–0.059	<0.001
Jumping	1.2	1.7	1.50	0.62–3.61	0.368
Facing forward	96.4	99.8	20.8	5.02–86.0	<0.001
Facing backward	3.0	0.2	0.070	0.017–0.279	<0.001
Standing	13.0	41.9	4.81	2.13–10.9	<0.001
Tail wagging	23.9	11.6	0.42	0.17–1.07	0.205
**Noise Level**	**Baseline Adjusted Mean (95% CI), dB**	**Intervention Adjusted Mean (95% CI), dB**	**Adjusted Difference, dB**	**95% CI**	**Holm-Adjusted *p*-Value**
Median dB	56.5 (52.4–60.6)	56.8 (52.7–60.8)	0.27	−5.48–6.03	0.918
Maximum dB	110.1 (107.7–112.5)	107.6 (105.1–110.1)	−2.49	−5.92–0.94	0.300

**Table 3:** Differences between Day 1 and Day 4 for Baseline and Intervention phases separately.

**Phase**	**Behaviour**	**Day 1** **Adjusted Probability (%)**	**Day 4** **Adjusted Probability (%)**	**OR (Day 4 vs. Day 1)**	**95% CI**	**Holm-Adjusted *p*-Value**	**Phase × Day Holm-Adjusted *p*-Value**
Baseline	Barking (short period)	3.8	4.9	1.33	0.39–4.49	1.000	1.000
	Barking (long period)	6.1	0.1	0.016	0.001–0.282	0.033	1.000
	Front of kennel	72.0	57.0	0.52	0.06–4.22	1.000	1.000
	Back of kennel	33.1	46.4	1.75	0.27–11.2	1.000	1.000
	Jumping	0.3	0.2	0.48	0.07–3.45	1.000	0.376
	Standing	19.4	8.5	0.39	0.09–1.60	1.000	1.000
	Tail wagging	22.3	11.5	0.45	0.08–2.62	1.000	0.911
Intervention	Barking (short period)	3.0	1.4	0.46	0.10–2.10	0.941	†
	Barking (long period)	0.6	< 0.1	0.025	0.001–1.08	0.274	†
	Front of kennel	99.3	99.8	3.64	0.17–78.5	0.941	†
	Back of kennel	1.0	0.3	0.27	0.02–4.46	0.941	†
	Jumping	0.2	1.3	7.96	1.11–57.1	0.234	†
	Standing	45.6	21.9	0.34	0.07–1.60	0.683	†
	Tail wagging	30.9	2.6	0.060	0.007–0.516	0.073	†
**Phase**	**Noise Level**	**Day 1 Adjusted Mean (95% CI), dB**	**Day 4 Adjusted Mean (95% CI), dB**	**Adjusted Day 4 − Day 1 Difference, dB**	**95% CI**	**Holm-Adjusted *p*-Value**	**Phase × Day Holm-Adjusted *p*-Value**
Baseline	Median dB	53.4 (37.4–69.3)	59.8 (43.9–75.8)	6.48	−16.1–29.1	0.857	0.760
	Maximum dB	112.1 (102.8–121.4)	109.0 (99.7–118.2)	−3.15	−16.3–10.0	0.857	0.760
Intervention	Median dB	53.2 (37.2–69.1)	53.3 (37.3–69.2)	0.10	−22.5–22.7	1.000	†
	Maximum dB	105.5 (96.2–114.8)	108.3 (99.0–117.6)	2.85	−10.3–16.0	1.000	†

† Phase × Day interaction *p*-values represent a single test comparing the Day 1-to-Day 4 change between Baseline and Intervention. For compact presentation, each interaction *p*-value is shown once, on the Baseline row for the corresponding outcome.

**Table 4:** Differences between Trial 1 and Trial 4 for Baseline and Intervention phases separately.

**Phase**	**Behaviour**	**Trial 1** **Adjusted Probability (%)**	**Trial 4** **Adjusted Probability (%)**	**OR (Trial 4 vs. Trial 1)**	**95% CI**	**Holm-Adjusted *p*-Value**	**Phase × Trial Holm-Adjusted *p*-Value**
Baseline	Barking (short period)	9.6	6.8	0.69	0.35–1.38	0.292	0.519
	Barking (long period)	12.5	1.0	0.071	0.025–0.202	<0.001	0.519
	Front of kennel	93.0	51.9	0.082	0.031–0.214	<0.001	0.014
	Back of kennel	8.3	49.8	10.92	4.36–27.34	<0.001	0.015
	Jumping	6.1	0.8	0.116	0.032–0.420	0.004	0.519
	Facing forward	97.6	91.8	0.27	0.12–0.62	0.006	0.519
	Facing backward	2.3	5.6	2.54	1.12–5.78	0.052	0.519
	Standing	30.3	4.5	0.110	0.054–0.224	<0.001	0.008
	Tail wagging	40.8	7.4	0.115	0.054–0.243	<0.001	0.167
Intervention	Barking (short period)	8.8	2.9	0.32	0.12–0.86	0.164	†
	Barking (long period)	1.5	0.4	0.25	0.077–0.806	0.163	†
	Front of kennel	99.6	99.6	0.98	0.29–3.25	1.000	†
	Back of kennel	0.6	0.6	1.03	0.32–3.35	1.000	†
	Jumping	4.0	1.8	0.44	0.16–1.23	0.706	†
	Facing forward	99.7	99.8	1.97	0.17–23.36	1.000	†
	Facing backward	0.2	0.1	0.48	0.035–6.46	1.000	†
	Standing	53.6	40.2	0.58	0.29–1.16	0.706	†
	Tail wagging	22.9	9.9	0.37	0.17–0.79	0.091	†
**Phase**	**Noise Level**	**Trial 1 Adjusted Mean (95% CI), dB**	**Trial 4 Adjusted Mean (95% CI), dB**	**Adjusted Trial 4 − Trial 1 Difference, dB**	**95% CI**	**Holm-Adjusted *p*-Value**	**Phase × Trial Holm-Adjusted *p*-Value**
Baseline	Median dB	59.6 (56.6–62.7)	55.4 (52.4–58.5)	−4.55	−8.02–−1.08	0.031	0.387
	Maximum dB	108.3 (104.4–112.4)	96.3 (92.5–100.4)	−11.62	−20.5–−2.69	0.031	0.387
Intervention	Median dB	58.1 (55.5–61.7)	56.8 (54.1–60.3)	−1.47	−4.96–2.01	0.550	†
	Maximum dB	105.3 (101.3–109.4)	101.1 (97.0–105.2)	−4.65	−13.6–4.31	0.550	†

† Phase × Trial interaction *p*-values represent a single test comparing the Trial 1-to-Trial 4 change between Baseline and Intervention. For compact presentation, each interaction *p*-value is shown once, on the Baseline row for the corresponding outcome.

**Table 5:** Differences between Trial 4 and Novel Person trial for Baseline and Intervention phases separately (second set of phases only).

**Phase**	**Behaviour**	**Trial 4** **Adjusted Probability (%)**	**Novel Person** **Adjusted Probability (%)**	**OR (Novel Person vs. Trial 4)**	**95% CI**	**Holm-Adjusted *p*-Value**	**Phase × Person Type Holm-Adjusted *p*-Value**
Baseline	Barking (short period)	0.8	1.4	1.67	0.21–13.31	0.630	1.000
	Front of kennel	75.1	99.2	40.22	2.13–760.98	0.041	0.237
	Back of kennel	25.6	1.9	0.057	0.006–0.501	0.040	0.237
	Standing	14.6	35.9	3.28	1.04–10.34	0.086	1.000
	Tail wagging	18.8	65.5	8.19	1.73–38.72	0.040	1.000
Intervention	Barking (short period)	2.8	4.7	1.73	0.31–9.58	1.000	†
	Front of kennel	99.8	99.7	0.79	0.08–7.72	1.000	†
	Back of kennel	0.8	0.9	1.20	0.17–8.71	1.000	†
	Standing	36.3	78.7	6.49	1.93–21.83	0.013	†
	Tail wagging	53.0	82.3	4.12	1.11–15.35	0.139	†
**Phase**	**Noise Level**	**Trial 4 Adjusted Mean (95% CI), dB**	**Novel Person Adjusted Mean (95% CI), dB**	**Adjusted Novel Person − Trial 4 Difference, dB**	**95% CI**	**Holm-Adjusted *p*-Value**	**Phase × Person Type Holm-Adjusted *p*-Value**
Baseline	Median dB	58.5 (52.4–64.6)	62.3 (56.2–68.4)	3.78	−9.17–16.7	0.789	1.000
	Maximum dB	99.1 (92.3–106.0)	96.7 (89.8–103.6)	−2.42	−33.4–28.6	0.815	1.000
Intervention	Median dB	61.6 (54.5–68.0)	63.0 (55.9–69.4)	0.84	−11.3–12.9	1.000	†
	Maximum dB	101.5 (93.0–108.2)	99.1 (90.6–105.7)	−1.84	−32.0–28.3	1.000	†

† Phase × Person Type interaction *p*-values represent a single test comparing the Trial 4-to-Novel Person change between Baseline and Intervention. For compact presentation, each interaction *p*-value is shown once, on the Baseline row for the corresponding outcome.

## Data Availability

Data are not publicly available but are available from the corresponding author upon reasonable request.

## Supplementary Materials

The following supporting information can be downloaded at https://www.mdpi.com/article/10.3390/ani16193016/s1, Table S1: Number of dogs contributing to behavioural observations by study set, phase, and day; Table S2: Sensitivity analysis comparing Baseline-to-Intervention differences between Set 1 and Set 2.

## References

[1] ASPCA U.S. Animal Shelter Statistics. https://www.aspca.org/helping-shelters-people-pets/us-animal-shelter-statistics.

[2] Powell L., Reinhard C., Satriale D., Morris M., Serpell J., Watson B. (2021). Characterizing Unsuccessful Animal Adoptions: Age and Breed Predict the Likelihood of Return, Reasons for Return and Post-Return Outcomes. Sci. Rep..

[3] Hennessy M. (1997). Plasma Cortisol Levels of Dogs at a County Animal Shelter. Physiol. Behav..

[4] Tuber D.S., Miller D.D., Caris K.A., Halter R., Linden F., Hennessy M.B. (1999). Dogs in Animal Shelters: Problems, Suggestions, and Needed Expertise. Psychol. Sci..

[5] Gilchrist R., Wynne C.D.L. (2025). Reducing Human-Directed Kennel Reactivity in Shelter-Housed Dogs. Pets.

[6] Wells D.L., Graham L., Hepper P.G. (2002). The Influence of Length of Time in a Rescue Shelter on the Behaviour of Kennelled Dogs. Anim. Welf..

[7] Sales G., Hubrecht R., Peyvandi A., Milligan S., Shield B. (1997). Noise in Dog Kennelling: Is Barking a Welfare Problem for Dogs?. Appl. Anim. Behav. Sci..

[8] Scheifele P., Martin D., Clark J.G., Kemper D., Wells J. (2012). Effect of Kennel Noise on Hearing in Dogs. Am. J. Vet. Res..

[9] Herron M.E., Kirby-Madden T.M., Lord L.K. (2014). Effects of Environmental Enrichment on the Behavior of Shelter Dogs. J. Am. Vet. Med. Assoc..

[10] Protopopova A., Wynne C.D.L. (2015). Improving In-kennel Presentation of Shelter Dogs through Response-dependent and Response-independent Treat Delivery. J. Appl. Behav. Anal..

[11] Baldan A.L., Ferreira B.L., Warisaia V., Feuerbacher E.N., Monticelli P.F., Gunter L.M. (2023). Reducing Barking in a Brazilian Animal Shelter: A Practical Intervention. Appl. Anim. Behav. Sci..

[12] Carrero J.C., Bennett S.L. (2025). Effect of Classical Counterconditioning (Quiet Kennel Exercise) on Barking in Sheltered Dogs. J. Vet. Behav..

[13] Payne S.W., Assemi K.S. (2017). An Evaluation of Respondent Conditioning Procedures to Decrease Barking in an Animal Shelter. Pet. Behav. Sci..

[14] Zurlinden S., Spano S., Griffith E., Bennett S. (2022). Impact of Classical Counterconditioning (Quiet Kennel Exercise) on Barking in Kenneled Dogs—A Pilot Study. Animals.

[15] Protopopova A., Mehrkam L.R., Boggess M.M., Wynne C.D.L. (2014). In-Kennel Behavior Predicts Length of Stay in Shelter Dogs. PLoS ONE.

[16] Hennessy M.B., Morris A., Linden F. (2006). Evaluation of the Effects of a Socialization Program in a Prison on Behavior and Pituitary–Adrenal Hormone Levels of Shelter Dogs. Appl. Anim. Behav. Sci..

[17] Wells D.L., Hepper P.G. (2000). The Influence of Environmental Change on the Behaviour of Sheltered Dogs. Appl. Anim. Behav. Sci..

[18] Powell L., Graham K., Grant L., Lenz O., Reinhard C.L., Yost H., Anderson E., Watson B. (2025). Insights into Shelter Dog Outcomes Using Behavioral and Biological Stress Markers. Physiol. Behav..

[19] Wells D.L. (1996). The Welfare of Dogs in an Animal Rescue Shelter. Ph.D. Thesis.

[20] McPeake K.J., Collins L.M., Zulch H., Mills D.S. (2021). Behavioural and Physiological Correlates of the Canine Frustration Questionnaire. Animals.

[21] Bremhorst A., Sutter N.A., Würbel H., Mills D.S., Riemer S. (2019). Differences in Facial Expressions during Positive Anticipation and Frustration in Dogs Awaiting a Reward. Sci. Rep..

[22] Bouton M.E., Broomer M.C. (2023). Learning to Stop Responding. Behav. Process..

[23] Nguyen H.A.T., Guo C., Homberg J.R. (2020). Cognitive Bias Under Adverse and Rewarding Conditions: A Systematic Review of Rodent Studies. Front. Behav. Neurosci..

[24] Roelofs S., Boleij H., Nordquist R.E., van der Staay F.J. (2016). Making Decisions under Ambiguity: Judgment Bias Tasks for Assessing Emotional State in Animals. Front. Behav. Neurosci..

[25] Bunzeck N., Guitart-Masip M., Dolan R.J., Düzel E. (2011). Contextual Novelty Modulates the Neural Dynamics of Reward Anticipation. J. Neurosci..

[26] Bunzeck N., Dayan P., Dolan R.J., Duzel E. (2010). A Common Mechanism for Adaptive Scaling of Reward and Novelty. Hum. Brain Mapp..

[27] Ogasawara T., Xu K., Snyder A.Z., Perlmutter J.S., Tu Z., Minamimoto T., Inoue K., Takada M., Monosov I.E. (2026). Temporal-Orbitofrontal Pathway Regulates Choices across Physical Reward and Visual Novelty. Neuron.

[28] Crump A., Arnott G., Bethell E.J. (2018). Affect-Driven Attention Biases as Animal Welfare Indicators: Review and Methods. Animals.

[29] Beesley C.H., Mills D.S. (2010). Effect of Kennel Door Design on Vocalization in Dogs. J. Vet. Behav..

