# Supplementary material for: Behavioural and Noise-Level Differences Associated with Response-Independent Treat Delivery in Shelter Dogs
Source: Animals (Basel). 2026 Sep 25;16(19):3016. doi: 10.3390/ani16193016 (PMC13663429; doi:10.3390/ani16193016)
Supplement: Supplementary file 1 [file animals-16-03016-s001.zip › animals-4524714-supplementary.pdf]

Supplementary Table S1. Number of dogs contributing to behavioural observations by study set, phase, and day

Set	Phase	Day 1	Day 2	Day 3	Day 4	Unique dogs across phase
1	Baseline	6	8	9	9	11
1	Intervention	6	5	5	4	6
2	Baseline	9	9	9	6	11
2	Intervention	9	10	6	11	13

Supplementary Table S2. Sensitivity analysis comparing Baseline-to-Intervention differences between Set 1 and Set 2

Outcome	Set 1 estimate (95% CI)	Set 2 estimate (95% CI)	Phase × Set *p*-value	Holm-adjusted *p*-value
Short barking	OR 0.42 (0.13–1.35)	OR 0.66 (0.26–1.68)	0.557	1.000
Continued barking	OR 0.04 (0.002–0.79)	OR 0.09 (0.01–0.76)	0.672	1.000
Front of kennel	OR 72.53 (9.09–578.82)	OR 93.22 (13.27–654.84)	0.857	1.000
Back of kennel	OR 0.02 (0.003–0.12)	OR 0.01 (0.002–0.08)	0.786	1.000
Jumping	OR 1.16 (0.36–3.74)	OR 2.10 (0.53–8.33)	0.528	1.000
Facing forward	OR 12.20 (2.03–73.32)	OR 45.79 (3.78–555.13)	0.397	1.000
Facing backward	OR 0.05 (0.007–0.42)	OR 0.09 (0.01–0.56)	0.717	1.000
Standing	OR 8.53 (2.29–31.74)	OR 3.31 (1.16–9.42)	0.272	1.000
Tail wagging	OR 0.22 (0.05–1.01)	OR 0.63 (0.19–2.03)	0.286	1.000
Median dB	+0.26 dB (−8.43 to 8.94)	+0.29 dB (−8.41 to 9.00)	0.995	0.995
Maximum dB	-6.59 dB (-11.04 to -2.14)	+1.83 dB (-2.74 to 6.39)	0.011	0.022
